# Use of a Generalized Additive Model to Investigate Key Abiotic Factors Affecting Microcystin Cellular Quotas in Heavy Bloom Areas of Lake Taihu

**DOI:** 10.1371/journal.pone.0032020

**Published:** 2012-02-23

**Authors:** Min Tao, Ping Xie, Jun Chen, Boqiang Qin, Dawen Zhang, Yuan Niu, Meng Zhang, Qing Wang, Laiyan Wu

**Affiliations:** 1 Donghu Experimental Station of Lake Ecosystems, State Key Laboratory of Freshwater Ecology and Biotechnology of China, Institute of Hydrobiology, Chinese Academy of Sciences, Wuhan, People's Republic of China; 2 Nanjing Institute of Geography and Limnology, Chinese Academy of Sciences, Nanjing, People's Republic of China; University of New South Wales, Australia

## Abstract

Lake Taihu is the third largest freshwater lake in China and is suffering from serious cyanobacterial blooms with the associated drinking water contamination by microcystin (MC) for millions of citizens. So far, most studies on MCs have been limited to two small bays, while systematic research on the whole lake is lacking. To explain the variations in MC concentrations during cyanobacterial bloom, a large-scale survey at 30 sites across the lake was conducted monthly in 2008. The health risks of MC exposure were high, especially in the northern area. Both *Microcystis* abundance and MC cellular quotas presented positive correlations with MC concentration in the bloom seasons, suggesting that the toxic risks during *Microcystis* proliferations were affected by variations in both *Microcystis* density and MC production per *Microcystis* cell. Use of a powerful predictive modeling tool named generalized additive model (GAM) helped visualize significant effects of abiotic factors related to carbon fixation and proliferation of *Microcystis* (conductivity, dissolved inorganic carbon (DIC), water temperature and pH) on MC cellular quotas from recruitment period of *Microcystis* to the bloom seasons, suggesting the possible use of these factors, in addition to *Microcystis* abundance, as warning signs to predict toxic events in the future. The interesting relationship between macrophytes and MC cellular quotas of *Microcystis* (i.e., high MC cellular quotas in the presence of macrophytes) needs further investigation.

## Introduction

Toxic cyanobacterial blooms in eutrophic lakes, rivers and reservoirs are encountered worldwide [1]–[3]. Microcystins (MCs) produced by some species of freshwater cyanobacteria are potent hepatotoxins and tumor promoters by inhibiting protein phosphatase types 1 and 2A [4], [5]. They can transfer via the food chain and accumulate in organisms [6], [7], causing poisoning even death of plants, invertebrates, fish, birds and mammals [8]–[11] in addition to effects on human health through chronic exposure [12], [13].

The MC toxic risks during cyanobacterial proliferations are determined by variations in both the abundance of toxic cyanobacterial strains and the production of MC by the toxic cells [14], [15]. The environment influences MCs indirectly by affecting the above two aspects. There have been many experimental and field studies to document the impact on MC production of various factors such as temperature, nutrients [16]–[19], light [17], [20], pH [21], [22], iron [23], xenobiotics [24], and predators [25]–[27], but the conclusions are sometimes different or even contradictory perhaps due to rather complex interactions of these factors in the field. It remains a great challenge to investigate how environmental factors interactively affect the toxicity of cyanobacteria. Thus, intensive and large-scale field surveys based on an effective model for data analysis are badly needed.

Generalized additive model (GAM) [28] is an extension of the generalized linear model. The advantage of the GAM is the adaptability for non-normally distributed variables. It is a flexible and effective technique for dealing with non-linear relationships between the response and the set of explanatory variables, and it is non-parametric generalization of multiple linear regression that is less restrictive in assumptions about the underlying distribution of data. The model assumes that the dependent variable is dependent on the univariate smooth terms of independent variables rather than independent variables themselves. The basic GAM model used took the following form:

E(Y|X_1_,X_2_,…,X_p_) = B_0_+S_1_(X_1_)+S_2_(X_2_)+…+S_p_(X_p_) where S_i_(X_i_), i = 1,2,…,p are nonparametric smooth functions (smoothing spline) for independent variable X_i_. The function S_i_ is estimated in a flexible manner and does not have to be nonlinear for all independent variables in GAM. The model is a useful and scientific tool applied in many scientific aspects [15], [29], [30].

Lake Taihu is the third largest freshwater lake in China, which historically has been beset by occurrences of cyanobacterial blooms dominated by *Microcystis* in warm seasons each year [31]. The coverage area of cyanobacterial blooms increased rapidly in recent years, posing serious threat to water supply for millions of inhabitants around the lake [32]. Water works located in the northern area supply drinking water to millions of residents of Wuxi city. Several field studies on MCs have been executed in Lake Taihu in recent years [33]–[37], but most of these studies were focused on two bays (Meiliang and Gonghu Bays) with simple description of seasonal changes of MCs, while systematic research on the whole-lake was still absent.

Mainly for these reasons, a systematic survey at 30 sites across the whole areas of Lake Taihu was conducted, and the spatiotemporal dynamics of MC concentrations, abundance and composition of major phytoplankton groups and various physicochemical parameters were monitored monthly from January to December 2008. MC cellular quotas which was calculated as the quotient obtained by dividing intracellular MCs concentration by *Microcystis* density presented MC-producing capability of *Microcystis* population. The main purpose of this study was to use GAM to investigate quantitative relationships between various environmental factors and MC cellular quotas from recruitment period of *Microcystis* to cyanobacterial bloom seasons, so as to clarify the possible mechanisms of environmental factors affecting MC-producing capability of *Microcystis* in the lake.

## Materials and Methods

### Ethics Statement

No specific permits were required for the described field studies. The location studied is not privately-owned or protected in any way and the field studies did not involve endangered or protected species.

### Study area

Lake Taihu (119°54′–120°36′N, 30°56′–31°33′E) in Jiangsu Province, is a subtropical, shallow, highly eutrophic freshwater lake with a surface area of 2338 km^2^, a mean depth of 1.89 m. It serves as an important resource for drinking water, irrigation, aquaculture, and industrial waters, in addition to being a popular recreational and tourist attraction. The occurrence of heavy cyanobacterial blooms in warm seasons has increased in frequency and intensity in recent years, which damages the function of the lake as a drinking water supply, posing a risk to public health [31].

### Sampling and analyzing

**Figure 1:**
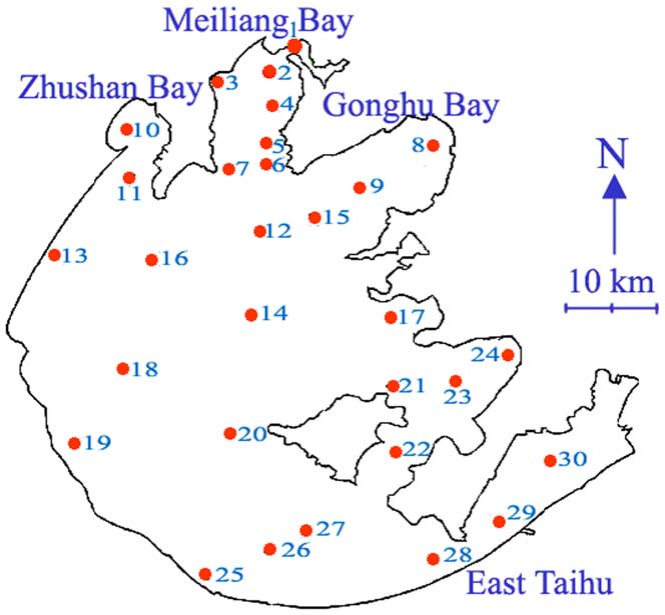
The sampling sites in Lake Taihu during the study period.

The lake was sampled at 30 sites (Fig. 1) from January to December, 2008. Sites 1–14 located in the northern area of the lake, where water was seriously polluted by human activities and was used as drinking water source, were sampled monthly. Water samples of other sites (sites 15–30) were collected quarterly. Each sample was a mixture collected from the top (0–0.5 m, surface water) and the bottom (0–0.5 m over sediment) of the water column with a 5 L Schindler sampler [38].

Values of water transparency and water depth were obtained *in situ*. Water temperature, pH, dissolved oxygen (DO) and conductivity were measured *in situ* with YSI Environmental Monitoring System 6600 (YSI Incorporated, Yellow Springs, OH, USA). Dissolved inorganic carbon (DIC) and dissolved organic carbon (DOC) were measured using a TOC Analyser (OI-1020A, OI Analytical, College Station, TX, USA), and some metal ions such as Na^+^ and K^+^ were analysed by ion chromatography (Dionex DX-100, Dionex Corporation, Sunnyvale, CA, USA).

Chemical parameters, including total nitrogen (TN), ammonia nitrogen (NH_4_-N), nitrate nitrogen (NO_3_-N), nitrite nitrogen (NO_2_-N), total phosphorus (TP), phosphate phosphorus (PO_4_-P), and chlorophyll a were measured for each sample according to the methods described by Greenberg *et al*. [39].

Water samples for identification of phytoplankton (1 L) were fixed *in situ* with acetic Lugol's solution [40]. In laboratory, each sample was concentrated to 50 ml after sedimentation for 48 h. Then 0.1 ml concentrated samples were counted using an Olympus microscope (BX50, Olympus, Tokyo, Japan) under magnification of ×400 after complete mixing. Colonial *Microcystis* cells were separated by using an ultrasonic crusher (JY88-II, Scientiz, Ningbo, Zhejiang, China), and then the single cells were counted. Phytoplankton species were identified with reference to the methods detailed by Hu and Wei [41] and John *et al*. [42].

MCs in lake water (1 L) were separated into extracellular MCs (toxins dissolved in water) and intracellular MCs (toxins in particulate) through filtering with a filter (Waterman GF/C, Whatman, Maidstone, Kent, UK). Filter films were extracted thrice in methanol (75%). The suspensions were centrifuged at a relative centrifugal force (RCF) of 24475× *g* (30 min at 4°C, Jouan KR22i, Jouan, Saint-Herblain, France) and the supernatant was diluted 1∶5 with distilled water. The MCs in distilled supernatant were directly concentrated on phase extraction cartridges (10 ml, C_18_, 500 mg), which were previously activated with 10 ml methanol (100%) and 10 ml distilled water. MCs were eluted from the cartridges with 10 ml methanol (100%) and then evaporated to dryness. The residue was dissolved in 100 µl distilled water and used for the qualitative and quantitative analysis of MCs. MC concentration was measured by using a Finnigan LC-MS system (Thermo Electron Corporation, San Jose, CA, USA) according to the methods described by Wang *et al*. [36].

### Statistical analyses

Regression analysis was performed using GAM, provided by PROC GAM procedure of the SAS software (release 9.1.3, SAS Institute incorporated, Cary, NC, USA) to assess the effects of environmental factors on MC-producing capability of *Microcystis* spp. in the recruitment, growth and proliferation phases of *Microcystis* bloom-forming (March to November). In order to better understand the underlying trend of any given factor, PROC GAM separates the linear trend from any general nonparametric trend during the fitting as well as in the final report. This makes it easy to determine whether the significance of a smoothing variable is associated with a simple linear trend or a more complicated pattern [43]–[45].

The model used the amount of intracellular MC concentration in each *Microcystis* cell (MC cellular quotas) as the dependent variable and abiotic factors such as temperature, pH, water depth, conductivity and nutrients as the independent variables. Zero values in MC cellular quotas were identified as outliers and excluded from the analysis. The “spline” function was used in MODEL statement to request an additive model using a cubic smoothing spline with four degrees of freedom by default for each environmental factor [43], [45]. Using conservative degrees of freedom in GAM is of benefit of avoiding over-fitting and lowers the computing cost. The F-statistic calculated from GAM vaguely indicated the relative strength of effect of an independent factor on dependent variable in the model. F-statistics were standardized to sum up to 100 within model. The product of standardized F-statistics (%) of each parameter and R-squares of the whole model presented the contribution of each parameter to MC production [15]. Factors with high significance levels (P<0.01) and accounting for the majority of the variations in MC production in the model were identified as key factors that have strong effects on MC production and discussed in detail. The combined effect of the linear and nonparametric contributions for each key factor was plotted using ODS Graphics statement [43], [45].

To test the conclusions of this study, the simplified model based on key factors was also applied to the data from previous studies in Lake Taihu, and the results were compared with results generated from the present *in situ* observations.

Other statistic analyses including Independent-samples T test and Spearman's correlation were carried out with SPSS version 13.0 for Windows (SPSS incorporated, Chicago, IL, USA).

## Results

### Environmental parameters

**Figure 2:**
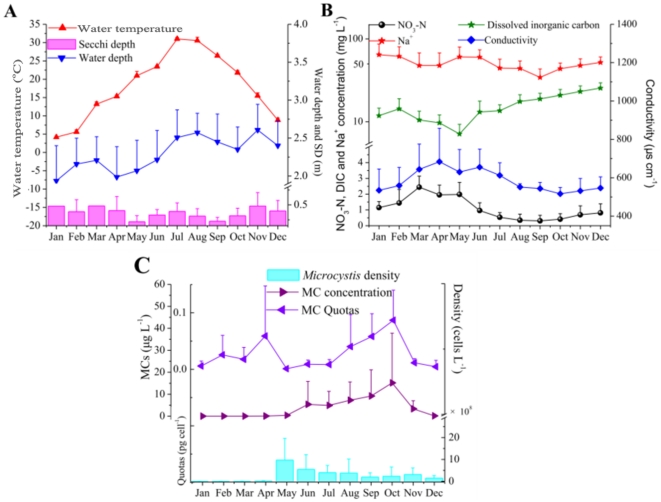
Seasonal variations of A) Water temperature, Water depth and Secchi depth; B) NO_3_-N, Na^+^, dissolved inorganic carbon (DIC) and conductivity and C) *Microcystis* density, MC concentration and MC cellular quotas in the northern area of Lake Taihu (values for each month are the mean value of fourteen sites).

Annual mean and ranges of the physical and chemical variables for the Lake Taihu in 2008 are presented in Table 1. Lake Taihu is an alkaline system, with pH values above 7.5 during the experimental period. Water temperature varied from 3.9 to 32.4°C and monthly means of water temperature in the northern area peaked in July and August (Table 1, Fig. 2A). Conductivity demonstrated an adverse seasonal variation trend to DIC: reached peaks in April before bloom broke out and experienced persistent decline until October except for June (Fig. 2 B).

**Table 1:** Mean and ranges of the environmental parameters during the study period of Lake Taihu.

	Northern area		Whole lake	
	Mean	Range	Mean	Range
*Microcystis* biomass (mg L^−1^)	18.9	0–330	19.2	0–330
Chlorophyll a (µg L^−1^)	0.023	0–0.22	0.021	0–0.22
Water depth (m)	2.3	1.2–5.5	2.3	1–5.5
Secchi depth (m)	0.37	0–1.6	0.41	0–2.1
Temperature (°C)	18.1	3.9–32.2	18.1	3.9–32.2
pH	8.25	7.52–9.64	8.25	7.49–9.64
Conductivity(µs cm^−1^)	584	390–1100	557	250–1100
DO (mg L^−1^)	9.15	0.1–16.29	9.26	0.1–16.29
Total nitrogen (mg L^−1^)	3.90	0.81–12.95	3.42	0.47–12.95
NH_4_-N (mg L^−1^)	0.94	0.06–6.15	0.77	0.06–6.15
NO_3_-N (mg L^−1^)	1.03	0.06–4.28	0.94	0.06–4.28
NO_2_-N (µg L^−1^)	60	0–400	47	0–400
Total phosphorus (mg L^−1^)	0.17	0.04–1.25	0.145	0.02–1.25
PO_4_-P (µg L^−1^)	21	3–126	18	1–126
TN∶TP ratio	26.6	7.5–59.6	26.4	7.5–63.7
Dissolved inorganic carbon (mg L^−1^)	15.5	3.9–34.5	14.5	3.6–34.5
Na^+^ (mg L^−1^)	50.5	21.1–125.4	48.6	11.9–125.4

**Figure 3:**
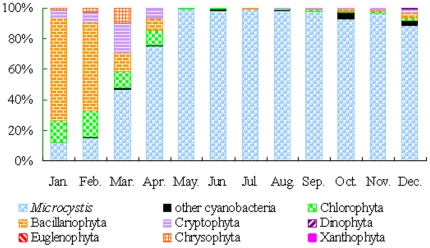
Temporal variation of phytoplankton density composition in the northern area of Lake Taihu.

**Figure 4:**
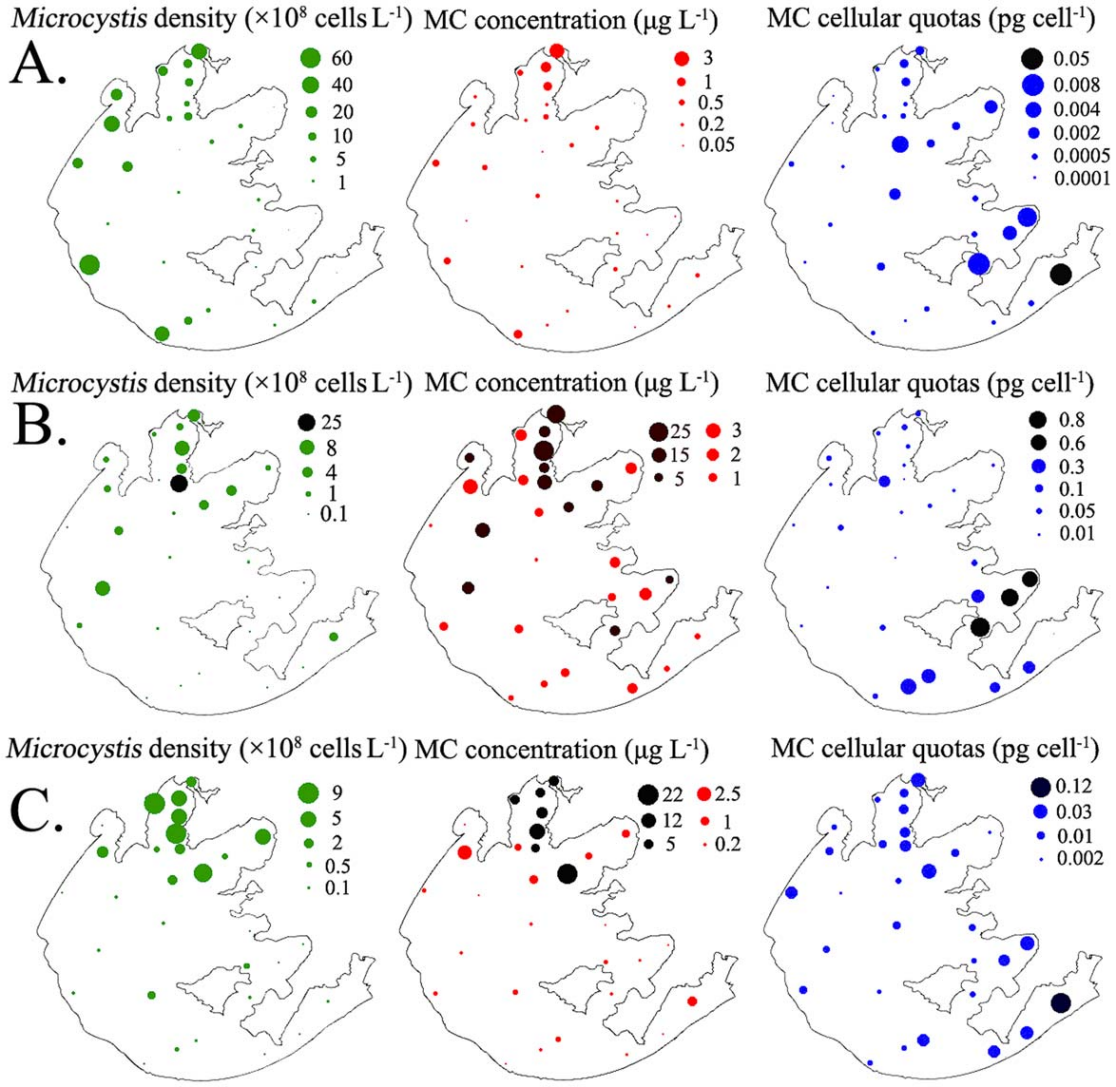
Spatial distribution of *Microcystis* density, MC concentration and MC cellular quotas in A) spring (May), B) summer (August) and C) autumn (November) of Lake Taihu.

A total of 87 phytoplankton taxa were recorded, with *Microcystis* spp. being the absolute dominant species in most months of the year. Temporal variation in phytoplankton abundance of various groups in the northern area was shown in Figure 3. Diatoms (Bacillariophyceae, mainly *Cyclotella* spp.), Cryptophyta and Chrysophyta jointly prevailed over the other groups only in winter and early spring. As the flourish of non-N-fixing *Microcystis* spp. in May, cyanobacteria became the absolutely advantageous taxa and maintained the superiority status in the remaining seasons. *Microcystis* spp. mainly contained *M. aeruginosa*, *M. flos-aquae*, *M. viridis* and *M. wesenbergii* in the study period. Spatial distribution of *Microcystis* biomass of Sites 1–30 from spring to autumn were demonstrated in Figure 4. *Microcystis* was abundant in the three northern bays and west littoral zones. Of which Meiliang bay was the most severely polluted area in Lake Taihu. Since spatial distribution of *Microcystis* revealed high risks in the northern area of Lake Taihu (Fig. 4), the present study is mainly focused on this area. Other potential MC-producing cyanobacteria (such as *Anabaena* spp. and *Oscillatoria* spp.) also multiplied during periods of *Microcystis* spp. dominance but accounted for a marginal part of cyanobacteria biomass (Fig. 3). Seasonal changes of *Microcystis* abundance in the northern area were shown in Figure 2C. Dramatic increase of *Microcystis* spp. gave rise to explosion of cyanobacteria density in May.

### Dynamics of MC concentration and MC cellular quotas

Spatial distribution of MCs in bloom seasons was shown in Figure 4, revealing a high health risk of MC exposure in the northern area, especially in Meiliang Bay (sites 1–7, 4.82 µg L^−1^ as a mean) where the MC concentrations were up to almost 14 times (Independent-samples T test; *P*<0.0001) higher than those in East Taihu (sites 28–30, 0.35 µg L^−1^ as mean). In the southern area, MC cellular quotas presented an adverse spatial distribution pattern to *Microcystis* abundance and MC concentration, which disclosed higher MC-producing capability of *Microcystis* (Fig. 4).

Seasonal variation of MCs in the northern area was shown in Figure 2C. MC concentration was much higher in summer and autumn than in the other seasons, and was at a low level in the first five months of 2008, but increased quickly from June to October when water temperature was above 20°C. It was obvious that variations of MC concentration did not always coincide with that of *Microcystis* abundance (Fig. 2C). *Microcystis* abundance and MC cellular quotas both presented positive correlations to MC concentration (Spearman's R = 0.46 and 0.63 respectively; P<0.0001) from recruitment period of *Microcystis* to bloom seasons (March to November), which indicated the importance of these two factors in prediction of toxic events. MC cellular quotas provide an estimate of mean MC-producing capability of *Microcystis* cells. The MC cellular quotas were higher in months when *Microcystis* cell abundance was relatively low (for example, in April and October) than in summer when *Microcystis* spp. bloomed (Fig. 2C).

### Results of GAM and test on previous data

Lake Taihu was an ideal system to study the complex mechanisms of various environmental parameters affecting MC-producing capability of *Microcystis* spp. for the naturally high levels of MCs observed in water and the high abundance of *Microcystis* (absolutely dominating the phytoplankton community) in the bloom seasons.

**Figure 5:**
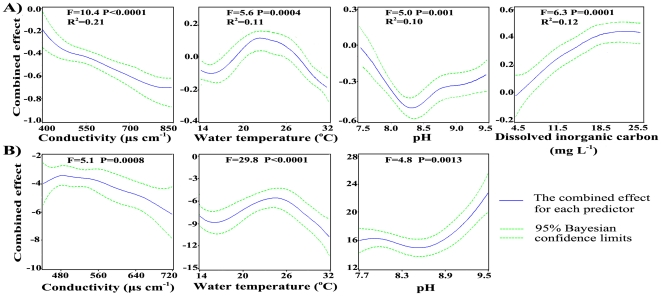
Plots showing the combined effect of the linear and nonparametric contributions for each important environmental factor on MC production by *Microcystis* spp. from recruitment period of *Microcystis* to bloom seasons of the GAMs run for A) the present study (R^2^ is the product of standardized F-statistics of each factor and R-squares of the whole model) and B) previous data.

From the results described above, the northern area was seriously polluted by toxic *Microcystis*. In consideration of the high risks and high *Microcystis* (absolutely dominating the phytoplankton community) abundance with high MC concentration, the present study was focused on the northern area (site 1–17) and GAM was used to investigate the key abiotic factors affecting MC-producing capability of *Microcystis*. Totally, all the abiotic environmental factors included in GAM could explain about 78% of the variations in MC cellular quotas. From the various factors, the highest weighted (P<0.01) four (conductivity, dissolved inorganic carbon, water temperature and pH), which accounted for the majority of the variations (54%) in MC cellular quotas in the study period (Fig. 5A), were finally selected to simplify our model (taking the costs and timeliness of monitoring into consideration). Nitrogen and phosphorus concentration had little effects on MC production of *Microcystis* spp. (data not shown).

In most field studies, complete data of conductivity, water temperature, pH and dissolved inorganic carbon (the key abiotic environmental factors confirmed in the present study) were usually lacking. Only two studies [36], [37] which offered relatively more information (three of the four factors mentioned here) were picked out to draw a comparison to the results obtained from the present study. The results showed that the three abiotic factors (water temperature, pH and conductivity) were all significant at 1% level in the GAM and could explain 61% of the variations in MC cellular quotas. Plots from the multivariate model showed that the overall trends of MC cellular quotas with the three factors in previous studies were similar to that in the present research (Fig. 5B), in spite of the differences in the details (Table 2).

**Table 2:** A comparison between the results of GAMs generated from present study and previous studies (Wang *et al*., 2010; Wilhelm *et al*., 2011).

	Parameter				
		Temperature	Conductivity	pH	DIC
Deviance explained by model	Present study	54%			
	Previous studies	61%			-
F	Present study	5.6	10.4	5.0	6.3
	Previous studies	29.8	5.1	4.8	-
P	Present study	0.0004	<0.0001	0.001	0.0001
	Previous studies	<0.0001	0.0008	0.0013	-
Pattern	Present study	Unimodal	Approximately linear	Curve	Approximately linear
	Previous studies	Unimodal	Approximately linear	Curve	-
Optimal conditions for MC production	Present study	21.5	Low value	-	High value
	Previous studies	24.5	Low value	-	-
Worst conditions for MC production	Present study	-	High value	8.3	Low value
	Previous studies	-	High value	8.5	-

## Discussion

### Temporal and spatial distribution of *Microcystis* spp. and MCs

The changes in abundance of *Microcystis* could not completely explain the fluctuations in MC concentration. Potentially MC-producing and non-MC-producing cells can coexist in natural cyanobacterial populations and the proportion of toxic cells can differ considerably over time during bloom season [46]–[48]. Many species of *Microcystis* spp. in Lake Taihu were potential MC producer like *M. aeruginosa*, *M. flos-aquae* and *M. viridis*, while another common species *M. wesenbergii* was reported to be non-toxic [49]. If a genotype can produce MCs, it should contain intact genes from the microcystin synthetase (mcy) gene cluster [50], [51]. Several studies have targeted the mcy gene cluster for the determination of relative abundance of MC-producing *Microcystis* cells in the total *Microcystis* population [50]–[52]. A study conducted in Meiliang Bay of Lake Taihu during the same period as the present research investigated the proportion of toxic *Microcystis* based on mcy gene (mcyA) and partial *Microcystis*-specific 16S rDNA sequence using real-time PCR. This research revealed shifts from non-toxic to toxic *Microcystis* strains from June to October, 2008 [53], which supported the result of increasing MC-producing capability in the northern area during this period in the present study. It seemed that the proportion of potentially toxic *Microcystis* cells increased with the development of bloom [35] when water temperature was above 20°C in Lake Taihu. It might be assumed that high MC cellular quotas in early spring are a result of the recruitment of highly toxic cells surviving the winter which preserved the mcy genotype composition from one year to the next [46].

The maximum mean concentration (15.2 µg L^−1^) and the maximum concentration (78.0 µg L^−1^) of MCs were both detected in October, significantly higher than those of *Microcystis* blooms in other regions of the world [54]–[56], revealed the severe contamination by MCs in Lake Taihu. In the present study, *Microcystis* abundance and MC concentration reached their peaks in different months, although they presented positive correlations. The possible explanation for this might be that compared to *Microcystis* density (Spearman's R = 0.46; P<0.0001), MC cellular quotas which represented the proportion of toxic cells had a closer relationship (Spearman's R = 0.63; P<0.0001) with MC concentration, so the maximum MC concentrations did not occur in the period of the heaviest algal blooms, but appeared in October when MC cellular quotas reached peak value simultaneously. Previous studies reported similar results [57], [58]. Seasonal variation of MC concentration indicated that the potential MC threat is present both during both bloom and non-bloom seasons in Lake Taihu, thus water safety in non-bloom seasons should also make an appeal.

Spatial distribution of *Microcystis* density and MC concentration in the bloom season warned of the high risks posed by MCs in the northern area. Meanwhile, it should be noticed that MC concentration in some sites of the eastern and western areas was also at a danger level in summer and autumn, despite the low density of *Microcystis* cells. The MC-producing capability of *Microcystis* in these areas was quite high. Interestingly, aquatic macrophytes could always be found in these sites during the bloom season. Phytoplankton and aquatic macrophytes are the primary producers in aquatic ecosystem; they compete intensely for various resources [59], and inhibit each other through secreting allelochemical such as microcystins and phenolic compounds [11], [60]–[62]. High MC production in this condition might be due to allelopathy and response of *Microcystis* to suboptimal conditions for growth resulted from competition with macrophytes.

### Abiotic factors influencing MC cellular quotas and test on previous studies

Environmental factors may affect the mean MC-producing capability of *Microcystis* spp. (MC cellular quotas) through the following two aspects, the proportion of potential MC-producing cells [63], [64] which possess the mcy gene cluster encoding MC synthesis [51] and expression level of mcy gene cluster [65], [66]. They may influence competitive ability of toxic and nontoxic *Microcystis* strains and the physiological condition of the toxic cells which are associated with MC production through their direct impact on cell division rate [14], [18], [20]. Various parameters including biotic and abiotic factors were reported to be regulating factors of MC production of cyanobacteria in field and laboratory (Table 3). The different results from previous studies and the complexity of interactions occurring among environmental factors in nature confirm the importance of very local conditions in the present research. In this study, we aimed to investigate the abiotic factors affecting MC cellular quotas and took no account of any biotic ones such as predators [25]–[27] in the model. All the parameters included in GAM accounted for 78% of the variations in MC cellular quotas. Theoretically, the biotic factors might account for the remaining 22% which could not be explained by the abiotic parameters included in the model.

**Table 3:** A comparison of the environmental factors affecting MC production of cyanobacteria from literatures and the present study.

Algae studied	Promoting factors	Inhibiting factors	Insignificant parameters	Reference
*Microcystis aeruginosa*	High light intensity	Low light intensity	Temperature and nutrients	Watanabe and Oishi, 1985
*Microcystis aeruginosa*	High iron concentration		Nutrients	Utkilen and Gjølme, 1995
*Microcystis aeruginosa*	High pH exceeded the value of 8.4			Jahnichen *et al*., 2001
*Microcystis aeruginosa*	Irradiances under the optimal point for growth	Irradiances higher than the optimal point for growth		Wiedner *et al*., 2003
*Microcystis aeruginosa*	Fish			Jang *et al*., 2004
*Microcystis aeruginosa*	Increasing intracellular inorganic carbon deficiency			Jahnichen *et al*., 2007
*Microcystis aeruginosa*	Nonylphenol of 0.05–0.5 mg/L			Wang *et al*., 2007
*Microcystis aeruginosa*	Infochemicals from zooplankton			Jang *et al*., 2008
*Microcystis viridis*	Both low and high pH (pH 7.0 and pH 9.2), lower light intensity	High light intensity	Temperature and nutrients	Song *et al*., 2007
*Microcystis* spp.	Increase nutrient loading			Vezie *et al*., 2002
*Microcystis* spp.	Optimum temperature (21.5°C,), high DIC and pH, low conductivity, competition with macrophytes		Nutrients	Present study
*Oscillatoria agardhii*	High nutrients concentration, low light intensity and optimal temperature	High light intensity		Sivonen, 1990
*Planktothrix* spp.	High cyanobacteria abundance, water depth		Temperature, irradiance and macronutrients	Halstvedt *et al*., 2008

Nitrogen and phosphorus concentration had little influence on MC production while factors related to carbon fixation and proliferation of *Microcystis* presented significant effects on MC cellular quotas. Perhaps nutrient loading in Lake Taihu (annual means of TN and TP were 2.98 and 0.123 mg L^−1^, respectively) was at a saturation level to MC production of *Microcystis*.

Conductivity which explained 21% of the variation of MC cellular quotas was the highest weighted parameters in the statistical model (Fig. 5A). It is a parameter related to the ability of electric conduction of water, and can indicate the ion concentration. *Microcystis* utilize various inorganic ions such as macronutrients and trace metal for growth. With the development of the population, available ions for growth decline and may become insufficient. Toxic cells have a competitive advantage over nontoxic ones under suboptimal conditions for growth [47], [67]. When environment conditions (for example, nutrients) were no longer well appropriate for growth, the proportion of toxic cells in *Microcystis* spp. increased and resulted in rise of MC production and MC concentration. The shifts from non-toxic to toxic *Microcystis* strains with the development of bloom when temperature was above 20°C might be due to deterioration of growth environment. Similarly, a test on previous data (Fig. 5B, Table 2) also showed a significant decrease in MC cellular quotas with conductivity (P = 0.0008).

Dissolved inorganic carbon (DIC) and pH could explain 12% and 10% of the changes in MC production, respectively (Fig. 5A). They were important parameters related to carbon fixation and proliferation of *Microcystis*. The photosynthesis of phytoplankton depletes dissolved carbon dioxide and increases pH and the concentration of dissolved oxygen in water. In such alkaline environments (low CO_2_/O_2_ ratios), cyanobacteria enable themselves to overwhelm other phytoplankton through establishing a carbon-concentrating mechanism (CCM) which adapts them to fluctuating inorganic carbon (C_i_) and O_2_ conditions to concentrate C_i_ more than 1,000-fold inside the cell [68]. HCO_3_^−^ transport system is one of the two functional elements composing CCM. Most commonly, HCO_3_^−^ is transported by an HCO_3_^−^ ATP binding cassette (ABC) transporter and two Na^+^-dependent HCO_3_^−^ transporters [69]. Consequently, Na^+^ is required for the active transport of C_i_ and can not be replaced by other monovalent metal ion such as K^+^ [70]. A negative correlation between Na^+^ concentration and DIC in *Microcystis* recruitment period and bloom seasons (Spearman's R = −0.237 *P* = 0.006) was found. The inscrutable decrease of Na^+^ limited uptake of C_i_ by *Microcystis*, caused C_i_ accumulation in water and posed relative deficiency of intracellular inorganic carbon (C_i,i_). MCs might be produced in response to a relative deficiency of C_i,i_ to enhance the efficiency of the adaptation of the photosynthetic apparatus to fluctuating inorganic carbon conditions in cyanobacterial cells [71]. This may help to explain the positive relationship between DIC and MC cellular quotas. Effects of pH presented a curve pattern with the lowest MC cellular quotas occurred in a moderate level (pH = 8.3 in the present study and pH = 8.5 in test on previous data, Fig. 5, Table 2). Because of the recruitment of highly toxic cells surviving the winter in early spring when pH level was low, MC cellular quotas were quite high [46], [47], [67]. It might be assumed that decreasing MC cellular quotas at increasing pH are due to the suitable conditions in company with multiplication and dominance in phytoplankton of *Microcystis* favored nontoxic cells over toxic ones. As the development of bloom, a gradually aggravating lack of free CO_2_ and decline in HCO_3_^−^ at increasing pH level when pH exceeded the value of 8.3 led to the enhance of MC production [22].

Temperature explained 11% of the variation of MC cellular quotas (Fig. 5A). MC cellular quotas changed with water temperature in a unimodal pattern with the maximum value occurred at 21.5°C. Test on previous studies showed a similar pattern with the maximum value at 24.5°C (Fig. 5B, Table 2). These results gave support to previous studies in both experiments [72] and field [73], suggesting that the optimal temperature for MC production by *M. aeruginosa* was between 20 and 25°C. As shown in Figure 2C, MC cellular quotas peaked in October when the mean water temperature was 21.8°C and changed with temperature as the predict pattern (Fig. 5A, Table 2) from June to November. However, MC-producing capability was quite low in May when the water temperature was theoretically optimal for MC production, which might be because of the small proportion of toxic cells.

The test on previous data shows that the overall trends of MC cellular quotas with the three factors (water temperature, conductivity and pH) in studies of Wang *et al*. [36] and Wilhelm *et al*. [37] were similar to that in the present research, in spite of the differences in details (Table 2). It should be noted that most of previous data applied in the model was from a study conducted in Gonghu Bay of Lake Taihu [36]. Compared with the northern area on which the present research focused, environmental condition in littoral Gonghu Bay with less water exchange was relatively stable. The different hydrographic conditions might be the cause of some differences between the two models. In spite of lack of DIC data, the test results from previous data reconfirmed the important roles of the other key factors obtained from the present study in regulation of MC production in Lake Taihu.

Variations of MCs are directly related to population dynamics of cyanobacteria [22] which include cell abundance, proportion and the physiological conditions of toxic cells. Although abundance of *Microcystis* cells are a traditionally indicator of toxic risks posed by MCs in many circumstances, the present study indicates that the changes in *Microcystis* abundance can not completely explain the fluctuations in MC concentration in Lake Taihu, and that water temperature, DIC, conductivity and pH are also important regulating factors.

### Conclusions

The health risks of MC exposure in Lake Taihu were high, especially in the northern area. *Microcystis* density and parameters affecting MC-producing capability of *Microcystis* were both important in predicting MC variation. As a powerful and scientific predictive modeling tool to discover the hidden pattern of predictors and improves the predictive performance, generalized additive model (GAM) was used to investigate quantitative relationships between abiotic environmental factors and MC cellular quotas from recruitment period of *Microcystis* to bloom seasons. The results of the model together with a test on previous data indicated that factors related to carbon fixation and proliferation of *Microcystis* (conductivity, DIC, water temperature and pH) presented significant correlations with MC cellular quotas, suggesting their possible use, in addition to *Microcystis* abundance, as warning signs to predict toxic events. The interesting relationship between macrophytes and MC cellular quotas of *Microcystis* needs further investigation.
